# The Prevalence of Hyperpalatable Baby Foods and Exposure During Infancy: A Preliminary Investigation

**DOI:** 10.3389/fpsyg.2021.614607

**Published:** 2021-04-13

**Authors:** Kai Ling Kong, Tera L. Fazzino, Kaitlyn M. Rohde, Katherine S. Morris

**Affiliations:** ^1^Baby Health Behavior Laboratory, Division of Health Services and Outcomes Research, Children's Mercy Research Institute, Children's Mercy Hospital, Kansas City, MO, United States; ^2^Department of Pediatrics, University of Missouri-Kansas City, Kansas City, MO, United States; ^3^Department of Pediatrics, University of Kansas Medical Center, Kansas City, KS, United States; ^4^Health Behavior and Technology Laboratory, Department of Psychology, University of Kansas, Lawrence, KS, United States; ^5^Cofrin Logan Center for Addiction Research and Treatment, University of Kansas, Lawrence, KS, United States; ^6^Division of Behavioral Medicine, Department of Pediatrics, Jacobs School of Medicine and Biomedical Sciences, University at Buffalo, Buffalo, NY, United States

**Keywords:** baby food, hyperpalatable, obesity, reward pathway, ingestive behaviors, infant dietary intakes

## Abstract

**Objective:** To characterize the prevalence of hyperpalatable foods (HPF) among baby foods in the U.S. and examine the prevalence of HPF exposure and consumption from both baby food and adult food sources among infants aged 9–15 months.

**Methods:** A U.S. baby food database as well as baby foods from three 24-h dietary recalls of 147 infants were used to identify baby foods as HPF per previous publication. HPF exposure was defined as intake of any HPF during the 3-day measurement period. To determine the extent of HFP consumption, % kilocalorie (kcal) intake from HPF was characterized.

**Results:** Only 12% of baby foods were HPF; however, nearly all participants (>90%) consumed HPF, primarily through exposure to adult foods. Younger infants (<12 months) consumed 38% [standard deviation (SD) = 23.6%] of their daily food kcal from HPF and older infants (≥12 months) consumed 52% (SD = 16.4%) of daily food kilocalorie from HPF. Most younger infants (68%) and older infants (88%) had repeated exposure to the same HPF across the measurement period.

**Conclusions:** The prevalence of HPF among baby foods in the U.S. is low. However, almost all infants were exposed to HPF, and HPF comprised a substantial percentage of daily food kilocalorie in infants' diets. Findings highlight the transition to solid food consumption during complimentary feeding period is a critical time for early HPF exposure.

## Introduction

Infancy is a sensitive developmental period and the most formative time for developing eating habits and food preferences that support growth needs. To this end, several investigations have shown that during the first year of life, food preferences are learned through repeated exposure to new foods (Schwartz et al., 2011; Nicklaus and Remy, 2013; Birch and Doub, 2014; Nicklaus, 2016). In a classic study by Sullivan and Birch, for example, feeding peas or green beans to 4-to-6-month-olds on 10 occasions, over approximately one month, led to significant increases in consumption of the vegetable over time (Sullivan and Birch, 1994). Relatedly, Maier et al. found that repeated exposure to a disliked vegetable (8 times in 16 days) early in the complementary feeding period resulted in intakes that were four-fold greater than the amounts the infants initially consumed (Maier et al., 2007). Thus, early exposure to healthy foods during the early feeding period may strongly influence infant feeding behavior.

In contrast to the evidence regarding healthy food exposure in infancy, early and repeated exposure to unhealthy foods among infants has not been well documented. Early exposure may adversely affect the establishment of food preferences and dietary intake patterns, which may have downstream consequences for obesity risk. For example, in the Melbourne Infant Feeding Activity and Nutrition Trial (InFANT) Program, consumption of nutrient-poor (sweetened beverages and cereals, sweet, and salty snacks) items at 9 months was strongly correlated with consumption of the same foods at 18 months (Lioret et al., 2013). Infants are highly sensitive to their nutritional environments; thus, early and repeated exposure to certain foods may dysregulate food reinforcement mechanisms and ingestive processes (Epstein et al., 2007). For example, hyperpalatable foods (HPF) contain combinations of palatability-inducing ingredients that can activate brain reward neurocircuitry and bypass physiological satiety mechanisms, leading to overeating despite feeling satiated (Fazzino et al., 2019). Early and repeated exposure to HPF during infancy may result in excessive activation of brain reward circuitry and reduced engagement of satiety mechanisms (Olszewski et al., 2019). With time, exposure to HPF may adversely affect the establishment of food preferences and drive infants to consume HPF, thereby heightening the risk of later obesity (Poti et al., 2017).

The availability of HPF among baby foods is unknown; thus, characterization of the prevalence of HPF among baby foods available in the U.S. food system is needed to understand the potential risk of exposure to HPF during infancy. Furthermore, no study has examined the degree to which infants in the U.S. may consume HPF during the complementary feeding period, a critical window in which food preferences may be most strongly established (Skinner et al., 2002). Specifically, while newborns start with a milk-based diet, they transition to complementary foods at ~6 months of age (this is accompanied by their discovery of foods with a variety of different smells, tastes, and textures), and finally to primarily table foods by the end of the second year of life. Exposure to HPF at any point within the first 2 years, but particularly during the early complementary feeding phases, may skew the development of food preferences and eating behaviors toward HPF. Therefore, limiting or avoiding exposure to HPF during infancy is likely ideal.

The objectives of our study were to: (1) examine the prevalence of HPF among baby foods available in the U.S. using the Nutrition Data System for Research, a database that is representative of the U.S. food system for the infant population, and (2) examine the prevalence of HPF exposure and consumption patterns among infants during the complementary feeding phase of 9–15 months of age. HPF were defined according to Fazzino et al. (2019).

## Methods

### Participants

The current study was a secondary analysis of data accrued from an ongoing longitudinal intervention (NCT02936284). Data used in the current study were obtained at the baseline measurement period. The sample consisted of 147 families with infants between 9 and 15 months of age. All families were recruited between 2017 and 2019. Due to the requirements of the intervention study, this sample excluded infants if: they were born preterm (<37 weeks' gestation), born with a low birth weight (<2,500 g), on any special diets, born with known medical problems, showed signs of developmental delays or disabilities, born to a mother who was <18 years at time of birth, born to a mother who smoked, used controlled substances, or consumed excessive alcohol during pregnancy, born in a high-risk pregnancy, and/or not a singleton.

### Procedures

Interested parents were screened via a telephone interview, and eligible parent-infant dyads were scheduled for a laboratory appointment. Parents were also sent links to study questionnaires before their appointment. The University's Institutional Review Board approved the study protocol and all participants provided written informed consent upon arrival to the laboratory. After parental consent was obtained, a packet of information regarding dietary data collection procedures (which included tips and frequently asked questions to guide the estimation of a child's food intakes) was given to the parents.

### Infant Dietary Collection

Parents of infants were contacted *via* a telephone call on three separate occasions (2 weekdays and 1 weekend), to collect three, 24-h dietary recalls for the infants; the procedure was a modified version from the Feeding Infants and Toddlers Study (FITS) (Anater et al., 2018). The telephone calls took place at a time deemed preferable by the participating parent. The recalls inquired about foods and beverages given by the parent, and if applicable, other caregivers (i.e., daycare providers). If the infant was still breastfeeding, the parent was instructed to keep track of their duration at the breast. Upon calling the parent, the research member asked if the infant had had a normal/healthy eating day 24 h prior; if not, then a new day to complete the recall was established. The research staff utilized a script to maintain protocol integrity. All research staff completed dietary recalls using the United States Department of Agriculture (USDA) Automated Multiple-Pass Method (Raper et al., 2004) and were extensively trained by a registered dietitian (MS/RD).

Once the telephone call for the dietary recall began, parents would be instructed to first give a quick list of everything their infant had consumed, involving no interruption from the research member. After receiving the quick list, the research member would look for any major eating gaps and inquire about possible beverages at those times. Following this, the research member would read back what they had written down and asked the parent to let them know if they wanted to modify anything. Upon completion of the first run-through, the research member would continue with more detailed questions about the infant's daily consumption, beginning with the first food on the list and asking open-ended questions to obtain further details when needed. If the parent struggled to estimate the amount of a food or beverage, the research member would refer them to the serving size guide in the handout packet. If something was unusual or unclear, the research member would seek clarity. At this point, the research member would ask about major eating gaps throughout the day again, and probe to see if anything was consumed. Before ending the call, information on supplements, medications, and the typicality of the days' eating were gathered.

### Data Processing: Baby Food Database and Intake Data

#### Baby Food Database

Data on baby foods available in the U.S. were obtained from Nutrition Data System for Research (NDSR; version 2019; Nutrition Coordinating Center, University of Minnesota, Minneapolis, MN). The NDSR software obtains information on the composition of foods and ingredients, which has been collected from the USDA National Nutrient Database for Standard Reference. In cases when information is missing, the developers of NDSR pull from other existing nutritional databases and scientific publications and employ necessary methodology in order to enhance validity of the values listed in the database. The baby food dataset is considered representative of the U.S. food system for babies and is one of the most comprehensive databases for infant food analysis. The dataset contained *n* = 1,084 total items as baby foods. Data were processed according to the definition for HPF (Fazzino et al., 2019). Specifically, liquids (i.e., infant formulas, juice, etc.) were removed from the dataset *n* (= 167) since the HPF definition only applies to solid foods. A total of *n* = 917 items was included in the analysis. For the majority of the food items (*n* = 853), a standard serving size was considered as the serving size indicated by the manufacturer (i.e., the portion of food used on the food's nutrition label), consistent with Fazzino et al. (2019). For a small minority of items, the total kcal per serving was listed as 0, which corresponded with a very small serving size (i.e., 1 puff had 0 kcal). To obtain a positive (greater than zero) kcal estimate, that was necessary for determination of hyper-palatability, the servings sizes of foods with less than 5 kcal per serving were increased to ¼ cup (*n* = 64). Increasing the serving size retained the original composition of the foods, while yielding a positive kcal count for analysis. The serving size adjustment did not alter the categorization of foods that met HPF criteria before vs. after the adjustment.

#### Intake Data

A total of *n* = 441 dietary recalls were available for *n* = 147 participants. All participants had 3 days of recalls in the final analysis. Across the recalls, infants consumed a total of *n* = 3,624 foods and *n* = 1,539 beverages, for a total of *n* = 5,163 items. Recalls were combined at the participant level for analysis. Of the foods consumed, *n* = 860 were baby foods and the remaining were foods typically consumed by adults (referred to herein as adult foods). Six foods were not included analyses, because a participant reported the infant consumed zero kcal. The HPF definition was applied to the *n* = 3,618 foods (non-liquids) available for analysis. The US Centers for Disease Control and Prevention (CDC) recommends breastfeeding or formula-feeding for a year before fully transitioning to table foods; thus, the sample was separated by younger infants (<12 months) and older infants (≥12 months) for analyses.

#### Application of HPF Criteria

Data were processed according to the HPF definition (Fazzino et al., 2019). For all food items in both the baby food and intake databases, variables for percent kilocalories (kcal) from fat, sugar, and carbohydrates (after removing sugar and fiber) were calculated. Percent sodium was calculated as percent sodium in grams per serving. HPF criteria were applied using the following metrics: (1) fat and sodium (> 25% kcal from fat, ≥ 0.30% sodium), (2) fat and simple sugars (> 20% kcal from fat, > 20% kcal from sugar), and (3) carbohydrate and sodium (> 40% kcal from carbohydrates, ≥ 0.20% sodium).

### Data Analysis

#### Baby Food Database

The prevalence of HPF overall in the baby food database was calculated by determining the number of food items that met HPF criteria divided by the total number of food items in the dataset. In addition, the prevalence of HPF among meal-based items and snacks were calculated separately for each category. Finally, the prevalence of HPF was calculated in light of the standardized Nutrition Data System for Research food category. Baby foods classified as miscellaneous or assigned to more than one food category (*n* = 241) were distributed into one of six final food categories by two co-authors, Morris, a registered dietitian, and Rohde, independently. Discrepancies between the two were resolved by Kong, the first author. The final outcomes were: fruits (*n* = 158), vegetables (*n* = 83), fruit and vegetable mixtures (*n* = 147), grain-based (*n* = 360), dairy and non-dairy alternatives (*n* = 69), and proteins (meat, fish, and other proteins) (*n* = 100).

#### Intake Data

Outcomes of interest were HPF exposure and pattern of HPF consumption. More specifically, the percentage of infants exposed to HPF was calculated as the total number of participants who consumed HPF (at least one HPF in their 3-day dietary recalls) divided by the total number of participants. To determine the source of exposure, the percentage of participants who consumed HPF as baby foods or adult foods was calculated. In addition, considering that repeated exposure to the same food may be strongly influential in the development of preferences for HPF, we determined the percentage of infants who consumed the same HPF on more than one day (in more than one diet recall). To examine the pattern of HPF intake among infants, percent kcal from HPF overall, and from each HPF group (fat and sodium, fat and simple sugars, and carbohydrate and sodium) was calculated.

Outcomes were reported for younger infants (<12 months) and older infants (≥12 months) separately. In line with the main focus of the study on solid food intake, primary analyses determined the percentage of daily HPF energy intake by calculating the total daily energy intake from HPF, divided by total daily energy intake from solid foods. However, because energy intake from milk may represent a substantial percentage of younger and older infants' intake, analyses were also conducted by calculating the percentage of daily energy intake from HPF out of total daily energy intake (milk + solid foods).

## Results

### Prevalence of HPF Among Baby Foods in the U.S. Food System

HPF comprised 12% (105/917) of total baby foods in the NDSR database. Of the HPF items, 26% (27/105) met criteria for the fat and sodium group, 52% (55/105) met criteria for the fat and simple sugars group, and 40% (42/105) met criteria for the carbohydrate and sodium group. Items were largely distinct, with 82% (86/105) of foods meeting criteria for a single food group (fat and sodium, fat and simple sugars, or carbohydrate and sodium).

The vast majority of items in the dataset (85%, 778/917) were meal-based items for breakfast, lunch, or dinner, such as purees and mixed dishes (e.g., chicken and vegetables). The remaining 15% (139/917) of items were snacks. HPF prevalence differed dramatically across meal and snack items. Specifically, 6% (47/778) of meal-based items were HPF. In contrast, 42% (58/139) of snack items were HPF. HPF snack items fell primarily into carbohydrate and sodium (47%, 27/58) and fat and simple sugars (43%, 25/58) groups. The snack items were primarily crackers (e.g., Earth's Best Organic Crunchin' Crackers–Cheddar) and snack bars (e.g., Plum Organics Jammy Sammy Snack Size Sandwich Bar–Peanut Butter and Grape).

Analyses of HPF prevalence by Nutrition Data System for Research food category revealed that HPF were differentially prevalent across categories (Table 1). Specifically, 25% (17/69) of foods in the dairy and non-dairy alternatives category met HPF criteria, with foods largely consisting of yogurt blends, and puddings. Similarly, the grain-based category had 21% (74/360) HPF prevalence and was comprised mainly of snacks such as crackers, biscuits/cookies, and snack bars. The proteins category had 10% (10/100) HPF prevalence, and consisted primarily of breaded proteins (e.g., fish nuggets) and protein-mixed dishes (e.g., Pasta Pick-Ups Beef and Tomato Ravioli). In contrast, no fruits met HPF criteria. One fruit and vegetable mixture (<1%, 1/147) met HPF criteria (Happy Tot Super Smart Organic Bananas, Mangos and Spinach + Coconut Milk) due to the elevated fat and sugar contents. Four percent (3/83) of vegetables category met criteria for HPF, all of which were breaded vegetable nuggets with elevated fat and sodium (e.g., Earth's Best Organic Gluten Free Baked Sweet Potato Nuggets).

**Table 1:** Percentage of hyperpalatable foods (HPF) among all baby food items in the Nutrition Data System for Research (*n* = 917).

**Category**	**HPF items identified (*n* = 105)**	**% HPF items as FSOD^***a***^**	**% HPF items as FS^***a***^**	**% HPF items as CSOD^***a***^**
Grain-based	74	21.0	31.4	34.3
Dairy and non-dairy alternatives	17	0.0	16.2	0.0
Proteins (i.e., meat, fish, etc.)	10	1.9	3.8	3.8
Vegetables	3	2.9	0.0	1.9
Fruits and vegetables mixtures	1	0.0	1.0	0.0
Fruits	0	0.0	0.0	0.0

a *Included food items that met the criteria for more than one hyperpalatable food group*.

*FSOD, fat and sodium; FS, fat and simple sugars; CSOD, carbohydrate and sodium*.

### HPF Exposure and Consumption Patterns Among Younger Infants (<12 Months) and Older Infants (≥12 Months) of Age

#### Descriptive Statistics

Characteristics of the infants and their mothers are presented in Table 2. Overall, the sample consisted of primarily highly educated families (≥ college graduates = 87.4%) of Caucasian race (77.6%). The mean and standard deviation (SD) for weight-for-length of the infants was 0.6 (0.9) z-score. Younger infants consumed a mean of 848.0 (SD = 214.0) kcal of total energy per day (milk + solid foods), of which 45% (SD = 13.7%) was energy from solid foods. Older infants consumed a mean of 985.1 (SD = 235.9) kcal of total energy per day (milk + solid foods), of which 67% (SD= 16.5%) was energy from solid foods. Both younger and older infants consumed the majority (≥60%) of their daily solid food energy intake from adult foods (younger infants: M = 60%, SD = 31.6%; older infants: M = 87%, SD = 15.3%) instead of baby foods.

**Table 2:** Participant characteristics (*n* = 147).

	**Mean (SD)**	***N* (%)**	**Range**
**Child**			
Sex, male		68 (46)	
Age, month	11.9 (1.9)		9.1–15.8
Race, Caucasian		114 (77.6)	
Refuse to answer		0 (0)	
Gestational age, weeks	39.4 (1.2)		37–43
Birth weight, kg	3.5 (0.5)		2.5–5.2
Weight-for-length z-score^a^	0.6 (0.9)		−1.7–3.1
Breastfeeding duration	8.1 (4.6)		0.5–15.8
<6 month		45 (30.8)	
≥ 6 month		101 (69.2)	
First introduction to solid foods	5.3 (1.0)		2.0–9.0
<4 month		5 (3.4)	
4–5 month		65 (43.6)	
≥ 6 month		76 (51.0)	
**Mother**			
Age, year	32.2 (4.3)		22.8–46.3
**Education level**			
Some college or below		22 (15.3)	
College graduate or higher		122 (87.4)	
Refuse to answer		0 (0)	
**Parity**			
Nulliparous		79 (54.1)	
Parous ≥ 1		67 (45.9)	
**Current BMI, kg/m^2^**	30.0 (7.7)		18.9–51.3
Normal weight		48 (33.3)	
Overweight/obese (≥ 25 BMI)		96 (64.4)	
**Household total income**			
< $30,000		13 (8.9)	
$30,000–$69,999		37 (25.2)	
$70,000–$109,999		55 (337.4)	
≥ $110,000		34 (23.1)	
Refuse to answer		8 (5.4)	

a *Calculated using the WHO growth charts*.

#### Exposure to HPF

**Figure 1:**
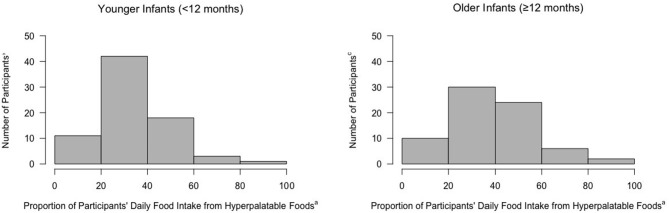
Proportion of daily food intake (kcal) from hyperpalatable foods among infants. ^a^Calculated as number of hyperpalatable foods consumed/total solid foods consumed per day. ^b^*n* = 75, Younger Infants. ^c^*n* = 72, Older Infants.

The vast majority of younger infants (91%; 68/75) and older infants (100%; 72/72) consumed HPF. Most younger infants (85% 64/75) and older infants (100%; 72/72) were exposed to HPF through adult foods. In contrast, 5% of younger infants (4/75) and 0% of older infants (0/72) were exposed to HPF solely through consuming baby foods. Exposure to HPF as a proportion of solid foods consumed per day is presented in Figure 1. The majority of both younger and older infants consumed between 20 and 60% of their daily foods from HPF (Figure 1). Regarding repeated HPF exposure, 68% of younger infants and 88% of older infants consumed the same HPF on more than one day.

#### Pattern of HPF Consumption

On average, younger infants consumed 38% (SD = 23.6%) of their daily solid food energy from HPF and older infants consumed 52% (SD = 16.4%) of daily solid food energy from HPF. Younger and older infants consumed the greatest percentage of HPF energy from fat and sodium foods, followed by carbohydrate and sodium foods, and fat and sugar foods (presented in Table 3). Examples of commonly consumed HPF from baby foods consisted of various crackers and puffs, yogurts, and cookies. Commonly consumed HPF from adult foods consisted of various cheeses (e.g., cheddar), breakfast foods (e.g., waffles, pancakes), and snacks (e.g., animal crackers, chips, and cereal). When considering total daily energy intake (milk + solid foods), younger infants consumed 19% (SD = 13.6%) of their total daily energy from HPF and older infants consumed 35% (SD = 14.5%) of their total daily energy from HPF.

**Table 3:** Percentage of daily caloric intake from hyperpalatable foods (HPF) among infants.

**HPF category**	**Younger infants (<12 months)**	**Older infants (≥12 months)**
	**M (SD) (%)**	**M (SD) (%)**
Energy intake from FSOD^a^	19 (19)	34 (15.1)
Energy intake from FS^a^	10 (11)	9 (8.5)
Energy intake from CSOD^a^	15 (15.5)	20 (12.4)

a *Calculated as energy intake of HPF/total energy intake from solid foods*.

*FSOD, food and sodium; FS, fat and simple sugars; CSOD, carbohydrate and sodium*.

## Discussion

The study sought to characterize the prevalence of hyperpalatable foods (HPF) among baby foods available in the U.S. food system, and to determine the degree to which infants are exposed to HPF within the first 15 months of life, a critical period for the development of food preferences and eating habits. Findings indicated that a minority (12%) of available baby foods in the U.S. met criteria for hyper-palatability, suggesting that for the most part, foods available to infants in the U.S. may not contain ingredients that are designed to artificially enhance palatability. However, analysis of infants' dietary intakes revealed that the vast majority (>90%) of younger infants (<12 months) and older infants (≥12 months) consumed HPF, primarily through exposure to adult foods. Furthermore, dietary energy intake from HPF was high and comprised over one third of daily solid food energy consumed among younger infants and over half of daily solid food energy consumed among older infants.

Our findings indicate that overall, infants may have a low risk for exposure to HPF when consuming available baby foods in the U.S. food system. This is encouraging since the recently published 2020–2025 Dietary Guidelines for Americans below 2 years of age recommends completely avoiding foods with added sugars and excessive salt, while opting for a variety of multicolored vegetables, legumes, fruit, whole grains, full-fat dairy, meat, eggs, seafood, nuts, seeds, soy products, and oils (exact quantities differ between 0 and 11 and 12 and 23 months). Here, the majority of baby foods available were meal-based items such as purees, which largely did not appear to be altered in a way that artificially enhances palatability. However, analyses did reveal substantial differences in HPF prevalence across types of baby foods, with almost half (42%) of snack items meeting criteria for HPF, vs. a small minority of meal-based items meeting HPF criteria (6%). Thus, a potential point of risk for HPF exposure may be when infants are fed baby snack foods, such as crackers and biscuits. However, in general, at the food system-level, the potential for exposure to HPF when consuming baby foods alone appears to be low. This finding is encouraging and suggests that when consuming *only* baby foods, infants may be able to avoid the intake of foods that can strongly influence their taste preferences and excessively activate brain reward neurocircuitry.

Despite the low prevalence of HPF among baby foods in the U.S., our findings indicate that almost all infants in the sample were exposed to HPF. Notably, the vast majority of infants were exposed to HPF when consuming adult foods, indicating that the transition from a milk-based diet to complementary feeding with solid foods may be a key point for HPF exposure. While the risk of exposure to HPF through adult foods is concerning, it is not entirely surprising within the context of the current food environment in the US. Our prior work has indicated that >60% of adult foods available in the US were HPF (Fazzino et al., 2019); thus, the early exposure risk in infancy is likely the result of living in an obesogenic food environment replete with HPF. However, despite the challenges in limiting HPF exposure, it is likely that preventing exposure to HPF among infants is likely ideal for facilitating healthy neural, physical, and psychological development. In this regard, evidence from imaging studies conducted primarily among adults is accumulating to suggest that consuming a food high in multiple palatability-inducing ingredients, such as an HPF, strongly activates brain regions involved in reward processing, motivation, and executive functioning (Gordon et al., 2020). Importantly, these same brain regions are activated during consumption of other highly rewarding stimuli, including drugs (Gordon et al., 2020). While it is unknown whether the findings of imaging studies conducted among adults translates to infants, it is likely that infants' brains are more sensitive to external stimuli (including food) than adults, given their rapid neurodevelopment. Thus, limiting exposure to HPF is likely ideal for healthy development.

Early exposure to HPF may have early negative effects on reward processing, food preferences, and eating behavior. Studies in utero, noteworthily, demonstrate that flavors from the mother's diet are capable of entering the amniotic sac and influencing an infant's food preferences, for better and for worse. Furthermore, during the complementary feeding period, individuals prenatally exposed to garlic, anise, and carrots showed greater liking and/or intakes of foods with those tastes (Mennella et al., 1995, 2001; Schaal et al., 2000). From experimental research in rats, however, consumption of highly palatable foods throughout pregnancy has led to offspring with increased preferences for fat, sugar, and salt (Bayol et al., 2007). Despite the inability to examine prenatal HPF exposure in our cohort of infants, regular, repeated exposure to HPF postnatally, in infancy, equally presents a major cause for concern. Thus, our finding that most infants in our sample already had repeated exposure to HPF through daily dietary intake is disturbing. Notably, HPF comprised 38 and 52% of daily food energy intake among younger and older infants, respectively, suggesting that daily exposure was substantial. Overall, the results highlight the transition to solid foods, particularly adult foods, as a key potential point for early exposure to HPF, as well as the development of regular HPF intake patterns. Our findings further highlight the need for the Dietary Guidelines for Americans below age 2 to focus on delaying the introduction of HPF, which may lead to overconsumption.

Early and habitual exposure to HPF, coupled with a genetic predisposition, may greatly increase the risk of developing obesity in childhood and later adulthood (Berthoud et al., 2011). Food is a primary reinforcer, and infants are innately motivated to eat for survival purposes. However, in today's obesogenic environment, HPF are readily available, and their overconsumption during infancy may set the stage for infants to develop childhood obesity (Gearhardt et al., 2011a,b). Infants have trivial room for nutrient-poor foods; therefore, every calorie counts and caregivers should strive to meet the nutritional needs of their infants without providing excess energy. Furthermore, the types of food offered by caregivers are important for forming early food preferences that can influence later health (Nicklaus and Remy, 2013). An advantage of the infancy period is that caregivers have full control over the home food environment and can be empowered to feed their infants in a way that supports energy balance. Thus, infants' dietary intakes should be aligned with their growth and developmental needs, which can be achieved by minimizing the introduction of and exposure to HPF at a young age.

This study had several limitations. First, infant intake data were reported by caregivers and thus may be susceptible to overestimation (Fisher et al., 2008). However, dietary recalls are still the best available method to date for measuring infant dietary intakes in free-living conditions. Second, the study sample was comprised of infants who were primarily from high socioeconomic status families, and thus our findings may not be generalizable to more diverse cohorts. This study should be replicated in low-income populations because they are more likely to struggle with adhering to healthful lifestyles due to limited resources. Finally, the definition for HPF was developed based on food data from studies conducted in adults. Thus, HPF criteria might only represent nutrient thresholds to enhance food palatability for adults and not infants. However, it is possible that the threshold needed to enhance food palatability for infants is likely lower than for adults; infants have limited exposure to solid foods and they have substantially more taste buds than adults, which may intensify a food's taste. Thus, further work is needed to determine whether other foods may be hyperpalatable for infants at a threshold that is lower than the threshold for HPF that has been identified for adults.

Despite the limitations, this study had many strengths, including assessment during a critical and narrow window of development (the complementary feeding period), the use of a quantitative definition of HPF to identify target foods for analysis, and the use of three, 24-h dietary recalls, which is the best available measure to estimate infant nutrient intakes (Ma et al., 2009). For the dietary recalls, parents were given thorough instructions and physical copies of dietary recall guides, and registered dietitians were trained to conduct nutrition analyses. Lastly, we performed our nutrition analysis using the research graded program, Nutrition Data System for Research software. This database is supremely comprehensive, consisting of over 18,000 foods (1,000 specifically being baby foods) and is continuously updated to maximize accuracy in data collections.

In conclusion, this study was the first to examine the prevalence of HPF among infant foods, and the extent to which infants are exposed to HPF during the complementary feeding period. Our findings highlight the complementary feeding period as a key period of risk for HPF exposure and the development of regular HPF consumption patterns. Larger, longer term observational studies on HPF consumption among infants and toddlers are needed to advance the field's understanding of the potential adverse effects of HPF intake on infant reward processing, eating behavior, and lifelong obesity risk. In addition, future prevention and intervention efforts should focus on delaying exposure to HPF during this critical period of development.

## Data Availability Statement

The raw data supporting the conclusions of this article will be made available by the authors, without undue reservation.

## Ethics Statement

The studies involving human participants were reviewed and approved by University at Buffalo IRB. Written informed consent to participate in this study was provided by the participants' legal guardian/next of kin.

## Author Contributions

KK initiated and developed the research question and study design, assisted with the analytic plan, and drafted the manuscript. TF initiated and developed the research question, led the analytic plan, and drafted the manuscript. KR contributed to the data analyses, results interpretation, and revision of the manuscript. KM contributed to the data collection, nutrition data analyses, results interpretation, and revision of the manuscript. All authors approve the submitted and final versions.

## Conflict of Interest

The authors declare that the research was conducted in the absence of any commercial or financial relationships that could be construed as a potential conflict of interest.

## Abbreviations

Kcal: kilocalorie
HPF: hyperpalatable food.
